# Assessing multidimensional complexity in home care: congruencies and discrepancies between patients and nurses

**DOI:** 10.1186/s12912-022-00942-x

**Published:** 2022-06-24

**Authors:** Catherine Busnel, Fanny Vallet, Eleni-Marina Ashikali, Catherine Ludwig

**Affiliations:** 1 Geneva Institution for Home Care and Assistance (imad), Avenue, Cardinal-Mermillod 36, CP 1731, 1227 Carouge, Switzerland; 2 Geneva School of Health Sciences, HES-SO, University of Applied Sciences and Arts Western Switzerland, Geneva, Switzerland

**Keywords:** Complexity, Person-centered care, Integrated care, Home care, Nurses

## Abstract

**Background:**

Person-centered care allows for the inclusion of the totality of a person’s needs and preferences, beyond just the clinical or medical aspect. This approach requires the development of tools to allow for the integration of the patient in his/her healthcare. Based on a 30-item tool developed for nurses to evaluate the complexity of home care situations (COMID), this study proposed a version for the patients (i.e. COMID-P). Both instruments were used, independently by nurses and patients, to rate the complexity of individual situations, in order to compare ratings.

**Methods:**

The COMID-P and the COMID were completed during the fraXity study at the patients’ homes, independently by patients (aged 65 and over) and nurses. Item-level and scale-level analyses were performed using, Kappa and McNemar tests, and intra-class correlation (ICC).

**Results:**

A total of 159 pairs of COMID and COMID-P ratings were retained for analyses. Results demonstrated a high degree of patient/nurse agreement for 12/30 items, a moderate agreement for 10/30 items, and a low degree of agreement for 7/30 items. The intra-class correlation between the COMID-P and the COMID was high (ICC= .826, 95%CI [.761-.873]).

**Conclusions:**

The results demonstrate that patients and nurses can assess complexity using tools that have comparable structural properties. They also reveal congruencies and discrepancies in scoring the components of complexity, highlighting the need of reaching consensus in designing care plans. Further work is needed to demonstrate the benefits of joint assessment in developing care plans that truly meet patients’ needs.

**Trial registration:**

The fraXity study was registered in ClinicalTrials.gov, NCT03883425, on March 20, 2019.

## Background

In Switzerland, recent policies fostering care-at-home, ambulatory care, and shortened hospital stays (also called the ambulatory “shift” or “switch”) [1] have led to an increased percentage of the population being able to grow old and be looked after within the home setting [2]. However, the increasing prevalence of co-morbidities, the array of problems possibly encountered in home care (e.g. medical, social, psychological), and the relative instability of the situations, lead to increased levels of complexity in home care [3]. This accumulation of issues forces us to rethink the approach to care in a broader perspective, taking into account the multiple cumulative factors around the patient’s situation. In this context, the patients are considered not with a “functional” approach, but as a whole, with all the biopsychosocial aspects of their life trajectory [4, 5].

### Person-centered care: concept, models and implications

Person-centered care allows for the inclusion of the different aspects of the person’s situation as well as his or her needs and preferences, beyond just the clinical or medical aspect [6]. Taking this global perspective in order to establish adapted and personalized care plans demands a strong collaborative participation from the patient, the informal caregivers, as well as health and social professionals [7, 8]. This requires considering the “patient” as a full-fledged actor in the health system, much like healthcare professionals [7]. The inclusion of patients in the care decisions that affect them, leads to changes in the culture of care among professionals and patients. In this perspective, the patients themselves should be involved to share their perception of their situation in all of its complexity. The implementation of this person-centered philosophy of care requires joint actions that are helpful in determining goals which are appropriate and acceptable to the patients [9, 10].

Although person-centered care approaches are valued by most professionals and have beneficial effects on care provision [11], their application in practice can be heterogeneous [12]. Based on the conceptual framework of person-centered care [5, 13–16], different aspects, such as a shared power and responsibility among all people involved, could also be taken into account. Over the past two decades, various models, which recognize the patient as an expert in the management of his or her illness and which allow for an active role in his/her care, have been developed, such as the Chronic Care Model [17] or the Montreal Model [18]. This dynamic of shared expertise between health professionals, informal caregivers and patients is changing the perspective toward a “patient-as-partner” approach in healthcare [19, 20]. To truly focus care on people, health professionals must take into account the resources and, often complex, needs of patients, in a culture of care and shared responsibility [21]. A person-centered approach establishes goals of care that are shared by all stakeholders and involves patients. This approach is not only a philosophy of care, but also requires the concrete integration of the patients’ vision [10] and participation in an explicit and joint understanding of health outcomes [22].

To this aim, specific measurement methods have been proposed to actively include patients in the evaluation of their own situations in terms of health outcomes (patient-reported outcome measures, or PROMs) [23, 24]. Originally, PROMs were developed to give patients a voice in assessing treatment effectiveness, but today they have broader applications. Notably, they are used by informed professionals to include patients’ perspectives in care design in order to foster shared decision-making. Thus, patient-reported measures and clinician-reported measures can be used to design care plans that respond to individual needs [25]. Patient-reported outcome measures, or PROMs can enhance the patients’ empowerment and their involvement in decision-making by giving them tools to better understand and to become aware of the several health and contextual aspects of their situations, as well as to structure and facilitate the expression of their perceptions and feelings [26]. The communication between the patient and the clinician can improve with the use of PROMs as these measures allow for a shared understanding of a given situation and because the patients’ perspective can be taken into account. Yet, to our knowledge, the use of the same instrument to collect patient and clinicain measures of a given situation is an exception, especially for specific PROMs serving care design.

### Complexity in home care practice: toward nurses’ assessments (COMID)

The shift from inpatient to ambulatory / home care witnessed in Switzerland over the last decade [1] has led to an increase in the prevalence of complex care situations in home care settings [27]. Complexity can be defined as a multidimensional concept involving interactions between biological, socioeconomic, cultural, environmental and behavioral forces as health determinants [28]. Complex situations require reinforced joint and collaborative interventions from different actors (e.g. patients, nurses, doctors, social workers) [29], who must determine together care plans for a given patient. This begins with sharing their evaluation of the situation. As recommended internationally, and imposed by Swiss law, organizations delivering care at home in Switzerland use the Resident Assessment Instrument Home Care (RAI-HC) for a comprehensive assessment of patient health needs [30] by nurses. Although the RAI-HC usually entails a wealth of additional clinical indicators, scales and decision support tools (e.g. Changes in Health, End-Stage Disease and Signs and Symptoms, CHESS [31], Detection of Indicators and Vulnerabilities for Emergency Room Trips, DIVERT [32]) it is not possible for the nurses to determine if a situation is complex on the sole basis of these scales. Therefore, to identify complexity in domiciliary practice, we recently proposed an instrument to help healthcare professionals assess it: the COMID [33], a questionnaire which reports nurses’ clinical judgments on different factors that may contribute to the complexity of a situation. The COMID goes beyond the factual elements given in the RAI because it demands that nurses position themselves and judge a given situation based on their global evaluation and knowledge of the situation. It helps them to identify and to analyze the elements that contribute to render a situation complex. The construction of the tool was based on existing conceptions of complexity [34] and adapted to the context of home care practice which brought forward six domains of complexity relevant in this particular context [33] : (1) medical factors, (2) socioeconomic factors, (3) mental circumstances, (4) behavioral factors, (5) instability circumstances, and (6) care network factors. These domains illustrate fields of activity interacting at the micro, meso, and macro levels, and integrate “the patient, his/her health, his/her care environment, the contextual conditions of care, the accessibility of care, the required care needs which were addressed/mobilized, and the care carried out in interdisciplinarity and interprofessionality.” [35] (p.13) (Translation from French). This assessment of complexity, which includes several bio-psycho-social, contextual, and care aspects of the situation of the person, contributes to a more wholesome consideration of the person in order to establish a personalized care plan.

### Complexity in home care practice: toward patient assessments

The COMID was designed to be used by nurses in home care, due to their role in coordination. However, it is not sufficient on its own to guarantee the promotion of a person-centered approach. While measures of complexity in home care integrate numerous aspects of the patients’ situations that are essential to propose individualized care plans, they must also take into account the patients’ perceptions in order to truly involve them in their care decisions. Active participation of the patients implies taking into account their own assessment of the situation, including identifying the elements that contribute to its complexity. Patients’ evaluations of their own situations provide important and unique information about their personal situation, and health needs [36, 37]. Patient-reported outcome measures are important complementary tools to traditional clinical indicators in defining care plans in a consensual manner, which then fosters adherence and improves quality care [38]. Patients’ assessments may be different from those of professionals [39]. Moreover, discrepancies may help to identify the points to be discussed in order to reach a consensus, and may, therefore, be of great value when used appropriately [40]. Yet, to our knowledge, no previous study has compared patient and nurse assessments on the basis of the same tool. This also applies to the assessment of complexity in home care, and it is precisely this gap that the present study aims to address.

## Method

### Research aim, design and setting

The aims of the study were to (1) develop a version of the COMID for the patients (i.e. COMID-P) and to (2) identify elements of divergence and convergence between patient and nurse responses on these two tools. The study is cross-sectional including two assessments of complexity for the same situation: one by the nurse (using the COMID) and one by the patient (using the COMID-P). Data was collected in the canton of Geneva, Switzerland, from April 2019 to November 2019, during the second wave of the “fraXity” study [41]. A total of 204 comprehensive health assessments were conducted at home, mimicking homecare setting.

### Participants and materials

Participants – hereafter referred to as “patients” – were people aged 65 and over, without major cognitive impairment, living at home and participating voluntarily in the study. After an interview led by a nurse, the COMID and COMID-P were independently completed. Of the 204 assessments were carried out, 201 (98%) comprised of both nurse and patient assessments and had five or less missing data. Among these, 159 (78%) provided complete data for both the COMID and COMID-P and were retained for analysis (listwise deletion method). They correspond to 159 patients (40 men: 25.2%, 119 women: 74.8%), living at home and aged 79.35 7±.95 years (mean ±standard deviation) at entry into the fraXity project (first wave). At the time of the interviews, 55 patients (34.6%) were receiving home assistance (e.g. household help, meal delivery), 51 patients (32.1%) were receiving home care (e.g. care from a nurse), with or without home assistance. Finally, 53 patients (33.3%) were not receiving any home services at all.

The nurses who collected the data were two men (aged 29 and 40) and two women (aged 29 and 34), hired as research nurses for the fraXity study. All four were registered nurses, with bachelor degrees in nursing (BSc), and with substantial experience in home care or intensive care. Two of the nurses also had a master’s degree (MSc). In the fraXity study, the nurses took part in the development of the material used aside from the standardized interRAI-HC and the COMID, which also included the COMID-P itself. The nurses were trained in data collection using all the study instruments, with a particular focus on the patient-centered approach and the use of PROMs. Weekly meetings aiming to guarantee the standardization of the procedures were held. The number of evaluations completed by each nurse was as follows: 7 (4.4%), 36 (22.6%), 49 (30.8%), and 67 (42.1%).

The COMID [42] is a validated questionnaire used to assess complexity. It includes 6 domains of multidimensional complexity: medical health, socio-economic, mental health, behavioral, instability, care professionals/system. Each domain is assessed using 5 items (for the full English version: https://comid.imad-ge.ch/Home/ComidEnglish). Items are coded in binary mode (no, not complex=0; yes, complex=1) and the total complexity score corresponds to the sum of the answers (range: 0 – 30). In order to allow patients to assess the complexity of their situation, the original COMID was adapted as a patient-reported outcome measure (PROM) for use by the patient: COMID-P (Table 1). The adaptation brought to the COMID-P consisted in rewording the instructions and the items. Special attention was given in using easy-to-understand language and instructions were written in a way that would ensure homogeneity across assessors. Aside from this, the COMID and the COMID-P are alike. In the present study, the internal consistency – calculated using Cronbach’s alpha – was α =.754 for COMID and α =.743 for COMID-P. The coefficients do not differ significantly (χ^2^ = .219, *p* = .640) [43], and both reflect acceptable internal consistency [44].

**Table 1:** Short content of the COMID-P in French (original) and in English (translation for understanding)

COMID-P French version	COMID-P English version
1. Facteurs de santé médicale	1. Medical health factor
1. a. Aujourd’hui, avez-vous plus que deux maladies chroniques ou un/des symptôme(s) inexpliqué(s) ?	1.a. Do you currently have several chronic diseases (more than 2) and/or unexplained symptoms
1. b. Avez-vous des douleurs chroniques ?	1.b. Are you suffering from chronic pain?
1. c. Avez-vous une allergie et/ou une intolérance à un médicament?	1.c. Do you have any allergies and/or drug intolerances?
1. d. Prenez-vous plus de cinq médicaments différents par semaine ?	1.d. Are you taking more than 5 different medications/drugs per week?
1. e. Avez-vous des troubles cognitifs ?	1.e. Do you have cognitive deficits?
2. Facteurs socio-économiques aggravant l’état de santé	2. Social and economic factors that worsen health status
2. a. Avez-vous des difficultés financières régulières ?	2.a. Do you have regular financial difficulties?
2. b. Avez-vous quelqu’un de votre entourage qui vous apporte une aide régulière et qui serait fatigué, stressé, ou en colère à propos de l’aide qu’il vous apporte ?	2.b. Do you have a relative or someone in your circle who helps you on a regular basis who is tired, stressed, or angry regarding the help they give you?
2. c. Chez le médecin et/ou pour des démarches administratives, avez-vous des difficultés à comprendre les informations qui vous sont destinées ?	2.c. At the doctor’s office and/or when doing administrative paperwork, during administrative procedures, do you find it difficult to understand the information provided to you?
2. d. Selon vous, êtes-vous isolé socialement ?	2.d. According to you, are you socially isolated?
2. e. Votre logement est-il inadapté ou y a-t-il des obstacles à la mobilité dans votre environnement ?	2.e. Is your housing inappropriate or are there barriers to your mobility within your environment?
3. Facteurs de santé mentale aggravant l’état de santé	3. Mental health factors that worsen health status
3. a. Etes-vous déprimé ou avez-vous des idées suicidaires ?	3.a. Are you depressed or have suicidal ideation?
3. b. Avez-vous des troubles psychiques ou une maladie psychiatrique ?	3.b. Do you have psychiatric disorders and/or mental disorders (delusions, hallucinations, etc.)?
3. c. Avez-vous une ou des addictions ou dépendances ?	3.c. Do you have any addictions?
3. d. Vous sentez-vous anxieux ou angoissé ?	3.d. Do you feel stressed or anxious?
3. e. Est-ce que votre état mental varie au cours de la journée ?	3.e. Does your mental state vary during the day?
4. Facteurs comportementaux du clients	4. Patient’s behavorial factor
4. a. Sollicitez-vous de manière récurrente vos proches, votre entourage (famille, amis, voisins) et/ou votre réseau de soins (professionnels de la santé (médecin, infirmière), autres) pour de l’aide et/ou des soins ?	4.a. Do you repeatedly solicit help and/or care from your family, friends, neighbors and/or your health care network (health care professionals (doctor, nurse), others)?
4. b. La communication avec votre réseau de soins (entourage, proches, professionnels de la santé (médecin, infirmière)) est-elle conflictuelle au sujet de votre prise en soin ?	4.b. Regarding your care plan, is the communication with your network (circle, relatives, healthcare) professionals (doctor/physician, nurse) ambivalent and/or conflicting?
4. c. Vous sentez-vous inquiet au sujet de votre santé ?	4.c. Do you feel worried about your health?
4. d. Etes-vous agressif envers vos proches, votre entourage ou les soignants ?	4.d. Are you aggressive towards your circle (family, friends) or towards health professionals?
4. e. Refusez-vous ou vous opposez-vous aux soins ?	4.e. Do you refuse or oppose care?
5 Facteurs d’instabilité	5. Factors of instability
5. a. Votre état de santé s’est-il dégradé ce dernier mois ?	5.a. Has your health deteriorated in the last month?
5. b. Votre capacité à être indépendant a-t-elle diminué au cours du dernier mois ?	5.b. Has your ability to be independent decreased in the last month?
5.c. Vivez-vous une période de transition, de stress ?	5.c. Are you going through a period of stress or of transition?
5. d. Avez-vous ressenti des changements concernant vos capacités cognitives ce dernier mois ?	5.d. Have you experienced/noticed any changes in your cognitive abilities in the past month?
5. e. Estimez-vous que l’évolution de votre santé est imprévisible ou instable ?	5.e. Do you feel that the evolution of your health is unpredictable or unstable?
6. Facteurs relatifs aux intervenants et système de soins	6. Factors related to care providers and care system
6. a. Y a-t-il plus de trois professionnels qui interviennent régulièrement dans votre prise en soins ?	6.a. Are there more than three professionals regularly involved in your care?
6. b. Estimez-vous que la communication avec les différents professionnels concernant votre prise en soins n’est pas optimale, pas suffisante, pas adéquate ?	6.b. Do you feel that the communication with the different professionals concerning your care is not optimal, not sufficient, not adequate?
6. c. Considérez-vous que votre prise en soins manque de cohérence ?	6.c. Do you consider that your care is lacking in coherence?
6. d. Avez-vous des problèmes avec votre assurance ?	6.d. Do you have issues/problems with your health insurance?
6. e. Estimez-vous que votre prise en soins est ressentie comme lourde émotionnellement ou physiquement par vous et/ou par votre réseau de soins.	6.e. Do you feel that your care is emotionally or physically burdensome to you and/or your caregivers?

The original questionnaires are available at https://comid.imad-ge.ch/

The COMID and COMID-P were completed by a question allowing to qualify the whole situation as simple or complex (“Do you consider this/your situation as simple or complex?” respectively), also coded in binary form (simple=0; complex=1).

### Procedure

Comprehensive health assessments were carried out at the patients’ home by the nurses. The Resident Assessment Instrument Home Care (interRAI-HC) [30], served to guide the interview. Complexity assessment was administered at the end, after the interRAI-HC. The nurse and patient rated complexity, with the COMID and the COMID-P respectively. The COMID-P was systematically proposed after the COMID to avoid the patient’s answers influencing those of the nurse. The COMID-P questionnaire was explained to each patient indicating the importance of giving their opinion on their own situation. Standardized instructions were provided by the nurses, trained to use the COMID-P. The use of alternative simplified wording in easy to understand language was part of the training, in order to anticipate literacy issues. Upon completion of the COMID and COMID-P questionnaires, nurses and patients responded to the global complexity question. The data were collected by means of paper questionnaires formatted with the EvaSys solution (Stat’Elite, Yens, Switzerland) for automatic document reading, and then exported to the statistical analysis software.

### Analysis

Analyses were conducted at the level of each of the 30 items and at the overall score level. At the item level, Kappa (κ) tests were performed to assess the degree of agreement between patients and nurses. McNemar tests were used to estimate differences in frequency of “yes” responses between patients and nurses. Kappa (κ) and McNemar tests were also performed on the global complexity judgment asked after the questionnaire. Concerning the global scale scores, intra-class correlation (ICC) and Student’s t-test for paired samples were used to estimate overall agreement and differences in the frequency of “yes” rating between nurses and patients. Finally, receiver operating characteristic (ROC) analyses and Youden’s J index were conducted to identify the threshold value (from 0 to 30) that distinguishes a simple situation from a complex situation, both for nurses and for patients.

For κ coefficients, the thresholds used to judge the degree of agreement are κ > .61 for strong agreement, .41 < κ < .60 for moderate agreement, and κ < .40 for weak agreement [45]. An ICC > .75 was considered to reflect a good agreement coefficient [46]. For McNemar’s and Student’s t-tests, a threshold of *p*<.001 was used to reject the null hypothesis and conclude a significant difference between patients and nurses.

## Results

### Analyses at the item level: inter-rater agreement and complexity rating

The results of the Kappa (κ) test assessing interrater agreement are reported in Table 2. Seven items had coefficients interpreted as revealing disagreement or low agreement (κ < .41). These were the items relating to cognitive deficits, resistance or opposition to care, acute change in cognitive abilities, partnership between the different actors, therapeutic incoherence, health insurance problems and emotional and/or physical burden. Moderate agreement (κ between .41 and .60) was found for the items: chronic diseases, chronic pain, low level of literacy, inadequate housing, anxiety or anguish, mental function varies over the day, recurring solicitations, ambivalent and/or conflictual communication, unpredictability of health status, and multiple care providers, as well as for the complementary question reflecting simple or complex situation. The strong to excellent agreements (κ >.60) were found for the items relating to allergies/drug intolerance, polymedication, financial difficulties, exhaustion of informal caregivers, social isolation, depression and/or suicidal ideation, psychiatric disease, addiction, anxiety or anguish, recent deterioration of health status perceived by the patient, change in degree of independence, transition period. The kappa related to items relating to aggression could not be assessed, due to the absence of “yes” responses for the patients (only 3 nurses reported “yes” answers).

**Table 2:** Number and rates of “yes” answers given by patients and nurses, degree of agreement, *p*-value coefficient of the Kappa test, *p*-value of the McNemar test for each item of the multidimensional complexity questionnaire and for the global situation assessment question

				Kappa test		McNemar’s test
Questionnaire items	Patients (COMID-P) “Yes % (n)	Nurses (COMID) “Yes % (n)	Responses in agreement % (n)	Coefficient	*p*-value	*p*-value
1.a. Chronic diseases	37.7 (60/159)	55.3 (88/159)	77.4 (123/159)	0.559 ^+^	<0.001	<0.001
1.b. Chronic pain	47.8 (76/159)	64.8 (103/159)	79.2 (126/159)	0.590 ^+^	<0.001	<0.001
1.c. Allergies/drug intolerances	29.6 (47/159)	27.7 (44/159)	96.9 (154/159)	0.923 ^++^	<0.001	0.375
1.d. Polymedication	24.5 (39/159)	28.9 (46/159)	94.3 (150/159)	0.856 ^++^	<0.001	0.039
1.e. Cognitive deficits	18.2 (29/159)	13.8 (22/159)	80.5 (128/159)	0.279	<0.001	0.281
2.a. Financial difficulties	6.9 (11/159)	8.8 (14/159)	98.1 (156/159)	0.870 ^++^	<0.001	0.250
2.b. Exhaustion of informal caregiver	7.5 (12/159)	10.7 (17/159)	96.9 (154/159)	0.811 ^++^	<0.001	0.063
2.c. Low level of literacy	5.0 (8/159)	5.7 (9/159)	95.6 (152/159)	0.565 ^+^	<0.001	1
2.d. Social isolation	11.3 (18/159)	13.2 (21/159)	96.9 (154/159)	0.854 ^++^	<0.001	0.375
2.e. Inadequate housing	6.3 (10/159)	13.2 (21/159)	91.8 (146/159)	0.542 ^+^	<0.001	0.003
3.a. Depression and/or suicidal ideation	8.2 (13/159)	9.4 (15/159)	96.2 (153/159)	0.765 ^++^	<0.001	0.687
3.b. Psychiatric diseases	0.6 (1/159)	1.3 (2/159)	99.4 (158/159)	0.664 ^++^	<0.001	1
3.c. Addiction	5.0 (8/159)	4.4 (7/159)	96.9 (154/159)	0.650 ^++^	<0.001	1
3.d. Anxiety or anguish	15.7 (25/159)	11.3 (18/159)	89.3 (142/159)	0.545 ^+^	<0.001	0.143
3.e. Mental function varies over the day	9.4 (15/159)	7.5 (12/159)	93.1 (148/159)	0.555 ^+^	<0.001	0.549
4.a. Recurring solicitations	6.9 (11/159)	7.5 (12/159)	93.1 (148/159)	0.485 ^+^	<0.001	1
4.b. Ambivalent and/or conflictual communication	3.8 (6/159)	5.7 (9/159)	95.6 (152/159)	0.511 ^+^	<0.001	0.453
4.c. Worries about symptoms	11.9 (19/159)	10.1 (16/159)	94.3 (150/159)	0.711 ^++^	<0.001	0.508
4.d. Aggressiveness	- (0/159)	1.9 (3/159)	98.1 (156/159)	-	-	0.250
4.e. Resistance or opposition to care	1.9 (3/159)	2.5 (4/159)	96.9 (154/159)	0.270	<0.001	1
5.a. Recent degradation of health status perceived by the patient	23.9 (38/159)	28.3 (45/159)	93.1 (148/159)	0.821 ^++^	<0.001	0.065
5.b. Change in degree of independance	9.4 (15/159)	8.8 (14/159)	98.1 (156/159)	0.886 ^++^	<0.001	1
5.c. Transition period	15.1 (24/159)	18.2 (29/159)	93.1 (148/159)	0.751 ^++^	<0.001	0.227
5.d. Acute change in cognitive abilities	3.8 (6/159)	1.9 (3/159)	95.6 (152/159)	0.202	0.007	0.453
5.e. Unpredictability of health status	17.0 (27/159)	22.0 (35/159)	86.2 (137/159)	0.561 ^+^	<0.001	0.134
6.a. Multiple care providers	8.2 (13/159)	7.5 (12/159)	93.1 (148/159)	0.523 ^+^	<0.001	1
6.b. Partnership between the different actors	9.4 (15/159)	1.3 (2/159)	90.6 (144/159)	0.098	0.048	0.001
6.c. Therapeutic incoherence	1.9 (3/159)	1.9 (3/159)	96.2 (153/159)	-0.019	0.808	1
6.d. Health insurance problems	5.0 (8/159)	1.3 (2/159)	95.0 (151/159)	0.184	0.003	0.070
6.e. Emotional and/or physical burden	3.8 (6/159)	10.1 (16/159)	88.9 (143/159)	0.230	0.001	0.021
Simple or complex situation? ^b^	16.6 (26/157)	22.3 (35/157)	81.8 (130/157)	0.454 ^+^	<0.001	0.124

^a^Interpretations of kappas: strong to excellent > 0.60 (identified in the table by ++), moderate between 0.41 and 0.60 (identified in the table by +), disagreement to low agreement < 0.40

^b^This analysis focuses on the 157 situations for which the questions were completed by the patient and the nurse

The results of the McNemar tests assessing differences in the rates of “yes” responses are also provided in Table 2. The results reveal that the complexity rating is comparable between patients and nurses, except for 6 of the items. The rate of complexity statements for the item related to the partnership between the different actors was significantly higher for patients. Nurses on the other hand, provided significantly higher rates of complex statements on items relating to chronic diseases, chronic pain, polymedication, inadequate housing and emotional and/or physical burden.

### Analyses for the total score: inter-rater agreement, complexity rating and complexity cut-off value

From the perspective of the scale as a whole, regarding the degree of agreement, the intra-class correlation calculated on the total score of COMID-P and COMID is ICC=.826 with a 95% confidence interval (CI) of [.761-.873], reflecting good agreement between patient and nurse ratings. In terms of overall mean score on their respective scales, patients had a significantly lower score (M=3.56, SD=3.147) than nurses (M=4.05, SD=3.253; [t(158)= 3.359, *p* =.001]).

The results of the ROC analyses revealed area under the curve (AUC) values of .830 (*p* < .001) for COMID-P and .898 (*p* < .001) for COMID. These values reach a threshold above .800, indicating excellent diagnostic accuracy [47, 48]. This means that the global complexity evaluation item is a good variable for discriminating between simple and complex responses. The Youden index, calculated to identify the threshold value at which a situation is considered, is J=0.710 for the COMID and J=0.602 for the COMID-P. In both cases, J corresponds to a tipping point at a score of 4.5. In practice, a score below 5 indicates a situation perceived as simple. A score equal to or higher than 5 indicates a situation perceived as complex. This result applies to both nurses and patients.

## Discussion

### Summary of results and implications for the practice

The aim of this study was to identify elements of divergence and convergence between patients and nurses on complexity using the COMID questionnaires. The COMID-P was developed for the present study and was administered to patients for the first time. As indicated by the high completion rate (201/204), with only minimal missing data, the COMID-P is a tool that can be used and completed by patients aged 65 years or older, who are free of major cognitive deficits. The COMID-P has a good internal consistency (α=.743) in the same range as the original COMID (α =.80) [49], and comparable to complexity self-assessment among inpatients (α =.78) [50, 51].

At a general level (total score), complexity ratings by patients and professionals substantially correlate, showing global agreement. This result can be explained in part by the fact that the complexity assessment was conducted after the interRAI assessment, as is done in clinical settings. Thus, the correlation between the COMID-P and the COMID should also be high in clinical contexts. It is interesting to note that the complexity score for patients was significantly lower than for nurses. Overall, patients rated their situation as less complex than did nurses, yet with an extremely modest difference (Mdiff=0.6), and a questionable clinical relevance. As compared to available results, the value of complexity assessed by nurses with the COMID (M=4.05, SD=3.25) in the present study is more than 2 points lower than the score reported in real clinical setting (M= 6.41, SD= 4.35) [49]. This difference could be explained by the composition of the sample: research volunteers without major health/cognitive issues in the present study, versus clinical home care patients in the corresponding published data. Finally, the results showed that for both patients and nurses a score of ≥ 5/30 identifies situations that are judged overall as complex.

At the item level, comparisons between patients and professionals revealed that 12 items showed high, 10 items moderate, and 7 items low agreement. Most items with low agreement are in the domain related to care (i.e. resistance or opposition to care, partnership between the different actors, therapeutic incoherence, health insurance problems, emotional and/or physical burden) or to cognition (i.e. cognitive deficits and acute change in cognitive abilities). Furthermore, patients reported significantly higher complexity than professionals on items related to partnership between the different actors. Conversely, nurses reported significantly more complexity than patients for the items of chronic diseases, chronic pain, polymedication, inadequate housing and emotional and/or physical burden. These results highlight that despite of an overall high agreement, patients and professionals have divergent views on certain components of complexity. In this study, the reasons for discrepancies are not documented, but in practice, it will be important to identify and address each of them, case by case, to reach shared and informed care decision. In clinical settings, comparing each item in terms of divergence, and convergence, allows to open the discussion between the actors to elaborate a therapeutic link reinforced in the understanding of the shared situation [23].

### Perspectives of the use of the COMID-P

The COMID-P allows, on the same basis as the COMID, to identify factors that can contribute to the complexity of a situation.

The comparison of the responses given by the patient and the nurse is a basis for discussion regarding the similarities or differences in the perception of the patient situation, as well as a means to gain insight into the other person’s perspective. From this point of view, the joint use of the COMID and the COMID-P appears to be a unique opportunity to actively include the patient in shared care decision making [23]. Completion of PROMs, such as the COMID-P, prompts patients to think about their health and enables them to raise issues with nurses [52, 53]. As such, the COMID-P could be routinely proposed in home care settings in order to help professionals to quickly identify points of agreement and disagreement from measures capturing different points of views. Joint health information enriches the collection of data that the professional himself/herself could not detect [54]. Taking into consideration the patient’s point of view is essential in a participatory and multidimensional approach, especially with patients with cumulative, unstable, highly fluctuating health difficulties and in environments that are not always suitable.

In addition, the results of the joint evaluation of the professional and the patient are particularly important for improving communication [55] and optimizing care. The joint use of COMID and COMID-P could contribute to a better patient/healthcare professional relationship, allowing patients to be even more active in the health decisions that affect them [56]. Providing quality care in complex situations requires good collaboration between all health professionals, informal caregivers and the patient, who is considered a full partner. Measurement is necessary to assess, promote and disseminate individualized care in order to be aligned with patients’ priorities. Such an approach requires a shared assessment on the different aspects of the situation [57]. The COMID-P allows to solicit the person’s expertise to apply a participatory care model as advocated by the Chronic Care Model [17] or the Montreal Model [18]. Therefore, the mobilization of tools common to patients and professionals should help to operationalize the person-centered philosophy in practice [10] by not only taking into account the patients’ perception, but also in developing their self-management capacities [18]. In this respect, integrating an assessment of complexity by the patients themselves contributes to the development of their capacities to analyze and take into account the factors contributing to their health [58], by providing them with a complete assessment grid similar to that used by health professionals. The COMID-P has a high potential to involve patients in care decisions. Being a short, accessible, easy–to-score, self-reported measure, the COMID-P has the characteristics of practical PROMs, as described by Kroenke and collaborators [59].

In practice, to use the COMID-P, the professional must guide without influencing the patient’s answers. Also, the nurse’s expertise is important to actively engage the patient in an informed and shared reflection on his/her care [60]. Training of professionals is important to understand the usefulness of a parallel and complementary assessment. This was the case in this study, and thus allowed nurses to support patients in a neutral manner. Recommendations for practice include complexity training, a person-centered approach, the use of person-reported outcomes, as well as integration of outcomes and perspectives into reasoning and development of goals of care.

## Conclusion

The focus of this study was to help identify the convergences and divergences in the evaluation of the complexity of home care situations between patients and professionals. The results show that 1) COMID-P and COMID can be used in parallel with an identical structure, 2) the process is feasible and 3) the data is clinically usable [61]. If complexity is a multidimensional accumulation of problems [42], the resolution of the difficulties encountered implies the requirement of communication and collaboration between the different actors, in a multidimensional approach in which the evaluation of patients and professionals is essential [38].

Both the agreements and disagreements identified in this study on patient and nurse assessments of complexity provide valuable insights for discussing and setting goals of care in which patients are fully engaged. In future practice, the convergence of the COMID-P and COMID results by items could be underlined to strengthen the therapeutic links, indicating a common vision of the situation by the patient and the nurse. To do this, the assessment of complexity is a first step towards a better understanding of the situation allowing a fair integration of the person’s (patient’s) point of view (person-centered outcomes) in his or her environment, a good inter-professional collaboration, and to propose targeted and shared care. Altogether, the study is, to our knowledge, the first opportunity to bring professionals and patients to use the same instrument to assess individual care needs. Moreover, the joint use of the COMID and the COMID-P is an innovative opportunity for a committed partnership between the patient and the nurse. As such, this study is an empirical demonstration of the patient-centered approach.

### Study status

The fraXity study is closed at the time of manuscript submission. The study was registered at ClinicalTrials.gov on March 20, 2019, with the identification number of NCT03883425.

## Data Availability

The dataset generated during the fraXity study are deposited at FORS/SWISSUbase for data sharing (https://doi.org/10.23662/FORS-DS-1256-1). The COMID and COMID-P questionnaires are available at imad (https://comid.imad-ge.ch/).

## Abbreviations

COMID: Multidimensional complexity for home care nursing practice (Complexité multidimensionnelle pour la pratique infirmière à domicile)
COMID-P: Multidimensional complexity for home care nursing practice, tool for patients (Complexité multidimensionnelle pour la pratique infirmière à domicile, version patients)
HES-SO: University of Applied Sciences and Arts Western Switzerland (Haute École Spécialisée de Suisse Occidentale)
imad: Geneva institution for homecare and assistance (institution genevoise de maintien à domicile)

## References

[1] Roth S, Pellegrini S (2015). Virage ambulatoire. Transfert ou expansion de l’offre de soins ?[Ambulatory switch. Transfer or expension of treatment offer ?] ( (Obsan Rapport N°68).

[2] Barber S, Lorenzoni L, Ong P (2019). Price setting and price regulation in health care: lessons for advancing Universal Health Coverage Geneva: World Health Organization, Organisation for Economic Co-operation and Development.

[3] Grant RW, Ashburner JM, Hong CS, Chang Y, Barry MJ, Atlas SJ (2011). Defining patient complexity from the primary care physician’s perspective: a cohort study. Ann Intern Med.

[4] Håkansson Eklund J, Holmström IK, Kumlin T, Kaminsky E, Skoglund K, Höglander J, Sundler AJ, Condén E, Summer Meranius M (2019). “Same same or different?” A review of reviews of person-centered and patient-centered care. Patient Educ Couns.

[5] Håkansson Eklund J, Holmström IK, Kumlin T, Kaminsky E, Skoglund K, Höglander J, Sundler AJ, Condén E, Summer Meranius M (2019). “Same same or different?” A review of reviews of person-centered and patient-centered care. Patient Educ Couns.

[6] The American Geriatrics Society Expert Panel on Person-Centered Care (2016). Person-Centered Care: A Definition and Essential Elements. J Am Geriatr Soc.

[7] World Health Organisation (2007). People-centred health care : technical papers : International Symposium on the People-centred Health Care : reorienting health systems in the 21st century, the Tokyo International Forum, 25 November 2007.

[8] World Health Organization : Framework for Action on Interprofessional Education and Collaborative Practice. Geneva; 2010. Available at: https://www.who.int/publications/i/item/framework-for-action-oninterprofessional-education-collaborative-practice. 21174039

[9] Think Local Act Personal (2013). A narrative for person-centred coordinated care.

[10] Abdelhalim R, Grudniewicz A, Kuluski K, Wodchis W (2019). Person-centered and integrated care: a discussion of concepts. Int J Integr Care.

[11] Burgers JS, van der Weijden T, Bischoff EWMA (2021). Challenges of Research on Person-Centered Care in General Practice: A Scoping Review. Front Med.

[12] Kogan AC, Wilber K, Mosqueda L (2016). Person-Centered Care for Older Adults with Chronic Conditions and Functional Impairment: A Systematic Literature Review. J Am Geriatr Soc.

[13] Hudon C, Fortin M, Haggerty JL, Lambert M, Poitras M-E (2011). Measuring patients’ perceptions of patient-centered care: a systematic review of tools for family medicine. Ann Fam Med.

[14] Stewart M (2001). Towards a global definition of patient centred care. BMJ (Clinical research ed).

[15] Robinson JH, Callister LC, Berry JA, Dearing KA (2008). Patient-centered care and adherence: definitions and applications to improve outcomes. J Am Acad Nurse Pract.

[16] Zhao J, Gao S, Wang J, Liu X, Hao Y (2016). Differentiation between two healthcare concepts: Person-centered and patient-centered care. Int J Nurs Sci.

[17] Bodenheimer T, Wagner EH, Grumbach K (2002). Improving primary care for patients with chronic illness. The Chronic Care Model. JAMA.

[18] Pomey M-P, Flora L, Karazivan P, Dumez V, Lebel P, Vanier M-C, Débarges B, Clavel N, Jouet E (2015). Le « Montreal model » : enjeux du partenariat relationnel entre patients et professionnels de la santé. Santé Publique.

[19] Karazivan P, Dumez V, Flora L, Pomey M-P, Del Grande C, Ghadiri DP, Fernandez N, Jouet E, Las Vergnas O, Lebel P (2015). The Patient-as-Partner Approach in Health Care: A Conceptual Framework for a Necessary Transition. Acad Med.

[20] Bodenheimer T, Wagner EH, Grumbach K (2002). Improving primary care for patients with chronic illness: The chronic care model, part 2. Jama.

[21] Bingham CO, Noonan VK, Auger C, Feldman DE, Ahmed S, Bartlett SJ (2017). Montreal Accord on Patient-Reported Outcomes (PROs) use series - Paper 4: patient-reported outcomes can inform clinical decision making in chronic care. J Clin Epidemiol.

[22] Wiercioch W, Nieuwlaat R, Dahm P, Iorio A, Mustafa RA, Neumann I, Rochwerg B, Manja V, Alonso-Coello P, Ortel TL (2021). Development and application of health outcome descriptors facilitated decision-making in the production of practice guidelines. J Clin Epidemiol.

[23] Porter I, Davey A, Gangannagaripalli J, Evans J, Bramwell C, Evans P, Gibbons C, Valderas JM (2021). Integrating Patient Reported Outcome Measures (PROMs) into routine nurse-led primary care for patients with multimorbidity: a feasibility and acceptability study. Health Qual Life Outcomes.

[24] Weldring T, Smith SMS (2013). Article Commentary: Patient-Reported Outcomes (PROs) and Patient-Reported Outcome Measures (PROMs). Health Serv Insights.

[25] Boyce MB, Browne JP, Greenhalgh J (2014). The experiences of professionals with using information from patient-reported outcome measures to improve the quality of healthcare: a systematic review of qualitative research. BMJ Qual Safe.

[26] Eriksen J, Bygholm A, Bertelsen P (2022). The association between patient-reported outcomes (PROs) and patient participation in chronic care: A scoping review. Patient Educ Couns.

[27] Baumann A, Wyss K (2021). The shift from inpatient care to outpatient care in Switzerland since 2017: Policy processes and the role of evidence. Health Policy.

[28] Bonizzoni E, Gussoni G, Agnelli G, Antonelli Incalzi R, Bonfanti M, Mastroianni F, Candela M, Franchi C, Frasson S, Greco A (2018). The complexity of patients hospitalized in Internal Medicine wards evaluated by FADOI-COMPLIMED score(s). A hypothetical approach. PLoS One.

[29] Karam M, Chouinard M-C, Poitras M-È, Couturier Y, Vedel I, Grgurevic N, Hudon C (2021). Nursing Care Coordination for Patients with Complex Needs in Primary Healthcare: A Scoping Review. Int J Integr Care.

[30] Morris JN, Fries BE, Bernabei R, Steel K, Ikegami N, Carpenter I, Gilgen R, DuPasquier J-N, Frijters D, Henrard J-C (2012). Services à domicile (SD) interRAI. Manuel de l’utilisateur et formulaire d’évaluation. Version 9.1, édition canadienne française.

[31] Hirdes JP, Frijters DH, Teare GF (2003). The MDS-CHESS scale: a new measure to predict mortality in institutionalized older people. J Am Geriatr Soc.

[32] Costa AP, Hirdes JP, Bell CM, Bronskill S, Heckman G, Mitchell L, Poss JW, Sinha S (2015). The DIVERT Scale: a metho to identify the probability of unplanned emergency department use among frail community dwelling seniors. J Am Geriatr Soc.

[33] Busnel C, Marjollet L, Perrier- Gros-Claude O (2018). [Complexity in home care: Development of an assessment tool dedicated to nurses and results of an acceptability study] Complexité des prises en soins à domicile : développement d’un outil d’évaluation infirmier et résultat d’une étude d’accepabilité. Revue Francophone Internationale de Recherche Infirmière.

[34] Loeb DF, Binswanger IA, Candrian C, Bayliss EA (2015). Primary care physician insights into a typology of the complex patient in primary care. Ann Fam Med.

[35] Busnel C, Ludwig C, Da Rocha Rodrigues MG (2020). La complexité dans la pratique infirmière : vers un nouveau cadre conceptuel dans les soins infirmiers. Rech Soins Infirm.

[36] Safford MM, Allison JJ, Kiefe CI (2007). Patient Complexity: More Than Comorbidity. The Vector Model of Complexity. J Gen Int Med.

[37] McGilton KS, Sorin-Peters R, Sidani S, Boscart V, Fox M, Rochon E (2012). Patient-centred communication intervention study to evaluate nurse-patient interactions in complex continuing care. BMC Geriatrics.

[38] Meadows KA (2011). Patient-reported outcome measures: an overview. Br J Community Nurs.

[39] Reading MJ, Merrill JA (2018). Converging and diverging needs between patients and providers who are collecting and using patient-generated health data: an integrative review. J Am Med Inform Assoc.

[40] Florin J, Ehrenberg A, Ehnfors M (2005). Patients’ and nurses’ perceptions of nursing problems in an acute care setting. J Adv Nurs.

[41] Ludwig C, Busnel C (2019). Protocol of a case-control longitudinal study (fraXity) assessing frailty and complexity among Swiss home service recipients using interRAI-HC assessments. BMC Geriatrics.

[42] Busnel C, Marjollet L, Perrier-Gros-Claude O (2018). Complexité des prises en soins à domicile : développement d’un outil d’évaluation infirmier et résultat d’une étude d’acceptabilité. Revue Francophone Internationale de Recherche Infirmière.

[43] Diedenhofen B (2016). Musch J: cocron: A web interface and R package for the statistical comparison of Cronbach’s alpha coefficients International Journal of Internet. Science.

[44] Chronbach LJ (1951). Coefficients alpha and the internal structure of tests. Psychometrika.

[45] Landis JR, Koch GG (1977). The measurement of observer agreement for categorical data. Biometrics.

[46] Koo TK, Li MY (2016). A Guideline of Selecting and Reporting Intraclass Correlation Coefficients for Reliability Research. J Chiropr Med.

[47] Lusted LB (1960). Logical analysis in roentgen diagnosis. Radiology.

[48] Lusted LB (1971). Signal detectability and medical decision making. Science.

[49] Vallet F, Busnel C, Ludwig C (2019). [Analysis of the reliability of a multidimensional complexity scale instrument (COMID) for home care nurses] Analyse de la fidélité d’un instrument d’évaluation de la complexité multidimensionnelle (COMID) pour les infirmières à domicile. Rech Soins Infirm.

[50] de Jonge P, Stiefel F (2003). Internal consistency of the INTERMED in patients with somatic diseases. J Psychosom Res.

[51] Peters LL, Boter H, Slaets JP, Buskens E (2013). Development and measurement properties of the self assessment version of the INTERMED for the elderly to assess case complexity. J Psychosom Res.

[52] Field J, Holmes M, Newell D (2019). PROMs data: can it be used to make decisions for individual patients? A narrative review. Patient Relat Outcome Meas.

[53] Greenhalgh J, Gooding K, Gibbons E, Dalkin S, Wright J, Valderas J, Black N (2018). How do patient reported outcome measures (PROMs) support clinician-patient communication and patient care? A realist synthesis. J Patient Rep Outcomes.

[54] Staniszewska S, Haywood KL, Brett J, Tutton L (2012). Patient and public involvement in patient-reported outcome measures: evolution not revolution. Patient.

[55] Hansen H, Pohontsch N, van den Bussche H, Scherer M, Schäfer I (2015). Reasons for disagreement regarding illnesses between older patients with multimorbidity and their GPs – a qualitative study. BMC Fam Pract.

[56] Romeyer H (2021). The Expert Patient in the Digital Age: Between Myth and Reality. Digital Health Communications.

[57] Naik AD, Catic A (2021). Achieving patient priorities: an alternative to patient-reported outcome measures (PROMs) for promoting patient-centred care. BMJ Qual Safe.

[58] Zhao J, Gao S, Wang J, Liu X, Hao Y (2016). Differentiation between two healthcare concepts: Person-centered and patient-centered care. Int J Nurs Sci.

[59] Kroenke K, Monahan PO, Kean J (2015). Pragmatic characteristics of patient-reported outcome measures are important for use in clinical practice. J Clin Epidemiol.

[60] Truglio-Londrigan M, Slyer JT (2018). Shared Decision-Making for Nursing Practice: An Integrative Review. Open Nurs J.

[61] Gibbons E, Black N, Fallowfield L, Newhouse R, Fitzpatrick R, Raine RFR, Barratt H (2016). Patient-reported outcome measures and the evaluation of services. Challenges, solutions and future directions in the evaluation of service innovations in health care and public health, vol. Essay 4.

